# Fluorinated TiO_2_ Hollow Spheres for Detecting Formaldehyde under UV Irradiation

**DOI:** 10.3390/ma17040904

**Published:** 2024-02-15

**Authors:** Jianwei Zhang, Baoyu Huang, Xinlei Li, Chao Yang, Wenzhuo Zhao, Xiuhua Xie, Nan Wang, Xiaogan Li

**Affiliations:** 1 School of Artificial Intelligence, Dalian University of Technology, Dalian 116024, China; jwzhang@dlut.edu.cn; 2 School of Integrated Circuits, Dalian University of Technology, Dalian 116024, China; huangby@dlut.edu.cn (B.H.); lixinlei96@163.com (X.L.); 13193718736@163.com (W.Z.); wang_nan@dlut.edu.cn (N.W.); 3 Beijing Research Institute of Telemetry, Beijing 100076, China; ycbj220@126.com; 4 State Key Laboratory of Luminescence and Applications, Changchun Institute of Optics, Fine Mechanics and Physics, Chinese Academy of Sciences, No. 3888 Dongnanhu Road, Changchun 130033, China; xiexh@ciomp.ac.cn

**Keywords:** fluorinated-TiO_2_, TiO_2_ hollow sphere, formaldehyde gas sensor, UV irradiation

## Abstract

The fluorinated titanium dioxide (F-TiO_2_) hollow spheres with varying F to Ti molar ratios were prepared by a simple one-step hydrothermal method followed by thermal processing. The diameter of the F-TiO_2_-0.3 hollow spheres with a nominal ratio of F:Ti = 0.3:1 was about 200–400 nm. Compared with the sensor based on pristine TiO_2_ sensing materials, the F-TiO_2_-0.3 sensor displayed an enhanced sensing performance toward gaseous formaldehyde (HCHO) vapor at room temperature under ultraviolet (UV) light irradiation. The F-TiO_2_-0.3 sensor demonstrated an approximately 18-fold enhanced response (1.56) compared to the pristine TiO_2_ sensor (0.085). The response and recovery times of the F-TiO_2_-0.3 sensor to 10 ppm HCHO were about 56 s and 64 s, respectively, and a limit-of-detection value of 0.5 ppm HCHO was estimated. The F-TiO_2_-0.3 sensor also demonstrated good repeatability and selectivity to HCHO gas under UV light irradiation. The outstanding HCHO gas-sensing properties of the F-TiO_2_-0.3 sensor were related to the following factors: the excitation effect caused by the UV light facilitated surface chemical reactions with analyte gas species; the hollow sphere structure provided sufficient active sites; and the surface fluoride (≡Ti−F) created additional chemisorption sites on the surface of the TiO_2_ material.

## 1. Introduction

Formaldehyde (HCHO), a hazardous and pungently odorous chemical compound, is extensively utilized as a versatile solvent in building materials and interior furnishing applications [1,2]. Therefore, volatilized formaldehyde gas has become the main source of indoor air pollution. Prolonged formaldehyde exposure may cause headaches, memory loss, chronic dermatitis, and respiratory diseases, posing appreciable health risks with potential carcinogenic and leukemogenic effects under severe conditions [3,4]. According to the regulations of the World Health Organization (WHO), the safety limit for long-term human exposure to HCHO is 80 ppb [5,6]. Therefore, there is an urgent need to develop sensors capable of reliable real-time monitoring of trace formaldehyde vapor to safeguard human health.

Faced with different usage needs in different places, researchers have designed and developed many gas sensors, including contact combustion sensors, electrochemical sensors, surface acoustic wave sensors, quartz resonance sensors, optical sensors, and semiconductor sensors [6,7,8,9,10,11,12]. Among them, electrochemical sensors, optical sensors, and semiconductor sensors are widely used. The optical gas sensors, with their advantages of non-contact and non-destructive measurement, were widely used in high-risk industries such as the petroleum and chemical industries and coal mines. In particular, nanoplasmonic devices, metasurface-based sensors, and surface plasmon resonance (SPR) sensors have also been used for gas detection due to their properties of room temperature operation, low detection limit, high sensitivity, online real-time detection, and miniaturization [13,14,15,16,17,18]. Furthermore, electrochemical sensors have also been widely used in the field of gas detection due to their high sensitivity, low detection limit, and real-time monitoring ability. However, electrochemical gas sensors still suffer from high cross-sensitivity issues. The commercial electrochemical formaldehyde sensor SFA30 (Sensirion) with low detection limits (20 ppb) is also sensitive to CO, NO, and NO_2_ gases and has been reported by Pei et al. [19]. In addition, compared with optical sensors and electrochemical sensors, semiconductor gas sensors are the most widely used gas sensors because of their advantages of high sensitivity, low cost, easy large-scale preparation, easy operation, and small size. Semiconductor gas sensors based on metal oxides have been extensively investigated by researchers over the last few decades. Unfortunately, traditional metal–oxide–semiconductor (MOS)-based gas-sensing materials typically necessitate elevated operational temperatures spanning 200–400 °C, which introduces problems of high energy consumption and low safety [20,21,22]. In addition, working at high temperatures for a long time may lead to the growth of metal oxide particles, making the sensing signal drift and shortening the service life.

Typical strategies, including noble metal decoration, heterojunction constructing, surface energetics tuning, and UV irradiation, have been employed in an attempt to moderate the requisite working temperatures of metal oxide-based gas sensors [22,23,24,25]. UV irradiation can increase the number of free carriers inside the sensing materials and promote the redox reaction of gas molecules occurring on the surface of sensing materials. Therefore, light excitation could replace traditional thermal excitation to enable room temperature gas detection [26]. In addition, photogenerated holes will also effectively facilitate the desorption process of the gas [27,28,29]. Therefore, the MOS-based gas sensors usually show better room temperature gas-sensing performance when excited by ultraviolet light. For metal oxide materials with excellent photoelectric properties such as ZnO, In_2_O_3_, TiO_2_, etc., the optimization of their gas-sensing performance by UV irradiation is particularly significant [30,31,32,33]. In particular, the TiO_2_-based sensor displayed good room temperature sensing performance for HCHO gas when excited by UV light [32,33,34,35,36]. The TiO_2_ microspheres-based sensor reported by Li et al. displayed high sensing response to sub-ppm sensitivity for formaldehyde gas under UV irradiation without external heating [33].

Additionally, according to the recent literature, the surface fluorination of sensing materials is also an efficacious approach to boost sensor performance [37,38]. Na et al. reported that the fluorinated porous carbon nanoweb layers had outstanding sensing performances for NH_3_ gas [37]. Compared with the sensor based on pristine graphene oxide (GO), the F-GO-based sensor prepared by Park et al. displayed enhanced sensing performance to NH_3_, which is due to the increase in the number of holes in graphene oxide after fluoridation [38]. It has also been reported that the ≡Ti–F group can effectively constrain the recombination rate of photogenerated electron hole pairs, which is beneficial for improving the sensing properties of TiO_2_ materials under UV irradiation [39,40,41,42].

Furthermore, the reported work shows that semiconductor oxide nanomaterials with hollow structures with high specific surface area are beneficial for the adsorption of gas molecules because of their sufficient active sites, which is conducive to improving the sensing performance of the sensor [24,33,43,44,45]. Hence, in this work, the fluorinated TiO_2_ (F-TiO_2_) hollow spheres with varying F to Ti molar ratios were synthesized to enhance the sensing performance of the TiO_2_-based gas sensor. Scanning electron microscopy (SEM), X-ray diffraction (XRD), transmission electron microscopy (TEM), and X-ray photoelectron spectroscopy (XPS) were used to characterize the morphology, structure, and chemical valence of the prepared materials to ensure the successful preparation of F-TiO_2_ hollow spheres. The HCHO-sensing performances of F-TiO_2_ hollow sphere sensors with different F content under UV irradiation were analyzed. Among them, the F-TiO_2_ hollow sphere (F-TiO_2_-0.3 with a nominal ratio of F:Ti = 0.3:1) sensor displayed the best sensing performance for HCHO gas. 

## 2. Experimental

### 2.1. Synthesis of the F-TiO_2_ Hollow Spheres

All chemical reagents used in this work were analysis grade and were purchased from Aladdin Chemical Reagents.

**Figure 1:**
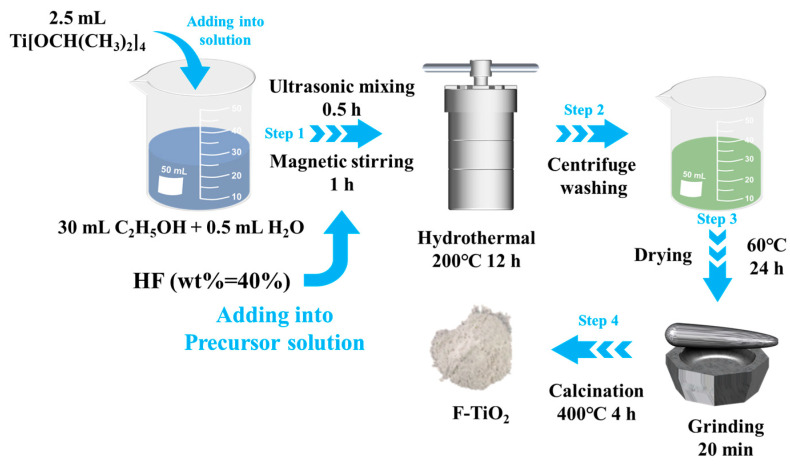
Chemical preparation process flow chart of the F-TiO_2_ materials.

The synthetic process of the F-TiO_2_ hollow spheres is displayed in Figure 1. Firstly, 2.5 mL isopropyl titanate was added into a mixture of 30 mL anhydrous ethanol solution and 0.5 mL deionized water and sonicated for 30 min to obtain a uniform precursor solution. Secondly, different volumes of hydrofluoric acid solutions (wt. 40%) were added to the above precursor solution and stirred continuously for 1 h to obtain a mixed solution with molar ratios of F and Ti elements of 0:1, 0.1:1, 0.2:1, 0.3:1, and 0.4:1, respectively. Thirdly, the obtained mixed solution was transferred to a hydrothermal reactor lined with polytetrafluoroethylene and heated at 200 °C for 12 h. Then, the reaction products obtained after centrifugation were washed with deionized water and ethanol several times, in turn, and placed in a drying oven at 60 °C for 24 h. Finally, the obtained solid products were heat treated at 400 °C for 4 h to obtain the fluorinated TiO_2_ hollow sphere material. For convenience, the obtained materials with different molar ratios of F and Ti elements were named as TiO_2_, F-TiO_2_-0.1, F-TiO_2_-0.2, F-TiO_2_-0.3, and F-TiO_2_-0.4.

### 2.2. Characterizations

The X-ray diffraction patterns were characterized by the diffractometer (XRD; Bruker D8 Advance, Germany) using Cu Ka radiation (0.15406 nm) in the range of 10° to 90° to investigate the crystal structure of the materials. The morphology of the sensing materials was characterized by a scanning electron microscope (SEM, S3000N, Hitachi, Japan) equipped with energy-dispersive X-ray spectroscopy (EDX) and a high-resolution transmission electron microscope (HRTEM, Tecnai G220 S-Twin) equipped with high-angle annular dark field scanning transmission electron microscopy (HAADF-STEM). The X-ray photoelectron spectrograms were obtained by the spectrometer (XPS, Thermo-ESCALAB-250XI) to investigate the element composition and valence states of the prepared materials. Electrochemical impedance spectroscopy (EIS), collected by high-precision electrochemical and impedance analyzers (Solartron, 1260A-1287A) via AC signal, had an amplitude of 500 mV and a frequency range of 10 Hz–1 MHz.

### 2.3. Sensor Fabrication and Electrical Measures

Five milligrams of the prepared sensing materials were placed in an agate mortar and ground with a small amount of deionized water for 20 min to obtain a uniform paste. The obtained paste was then dropped on the interdigital electrode displayed in Figure S1, followed by drying at 60 °C for 10 h to prepare a chemiresistive-type gas sensor based on the F-TiO_2_ hollow spheres. In this work, the response value is defined as $S=\frac{\left|R_{a}-R_{g}\right|}{R_{g}}\cdot 100\%$, where *R_g_* is the resistance of the sensor in the reducing target gas and *R_a_* is the resistance of the sensor in air. Response and recovery time are defined by the time required for the sensor’s resistance to reach 90% of its change value.

Figure S1 shows the static test system used to test the gas sensor’s performance. The testing system consists of four components, including a 50 L sealed testing chamber made of acrylic sheet, a computer, a data recorder (KEYSIGHT 34972A) as the data acquisition unit, and a power supply (KEYSIGHT E36311A DC). The data acquisition unit collects the sensor’s resistance value in real time, and the computer automatically controls the collection and storage of data. A UV LED light with a wavelength of 365 nm and an energy density of 2.5 mW/cm^2^ was used as a UV irradiation source. Additionally, the DC power supply also applies a rated DC voltage to the UV lamp to provide the UV light conditions required for the test. The UV light remained on throughout the testing process. A detailed test procedure for the sensor is described in the Supplementary Materials (see Figures S1 and S2 and Table S1).

## 3. Results and Discussion

### 3.1. Morphological and Structural Characteristics of Sensing Materials

**Figure 2:**
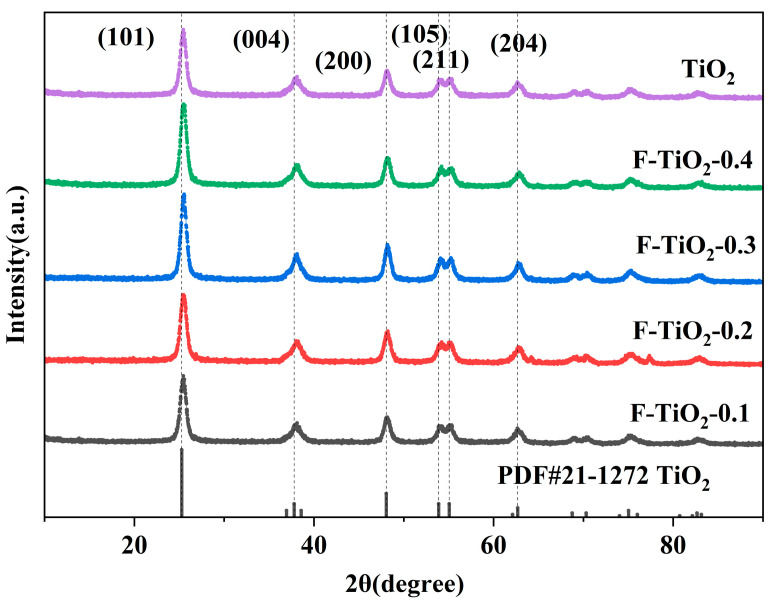
XRD spectra of F-TiO_2_ hollow spheres with different fluorine–titanium ratios.

Figure 2 exhibits the XRD diffraction patterns of synthesized pristine TiO_2_ and F-TiO_2_ hollow sphere materials with varying fluorine content. The diffraction peaks of all the F-TiO_2_ hollow sphere materials with different fluorine contents are consistent with those of the pristine TiO_2_. The significant peaks at 25.3°, 37.8°, 48.4°, 53.9°, 55.1°, and 62.7° correspond to the crystal planes of anatase-phase TiO_2_ (JCPDS No.21-1272) (101), (004), (200), (105), (211), and (204), respectively. All the diffraction peaks in the XRD patterns of the F-TiO_2_ material belong to TiO_2_ crystal, indicating that the fluorination process did not change the structure of TiO_2_.

**Figure 3:**
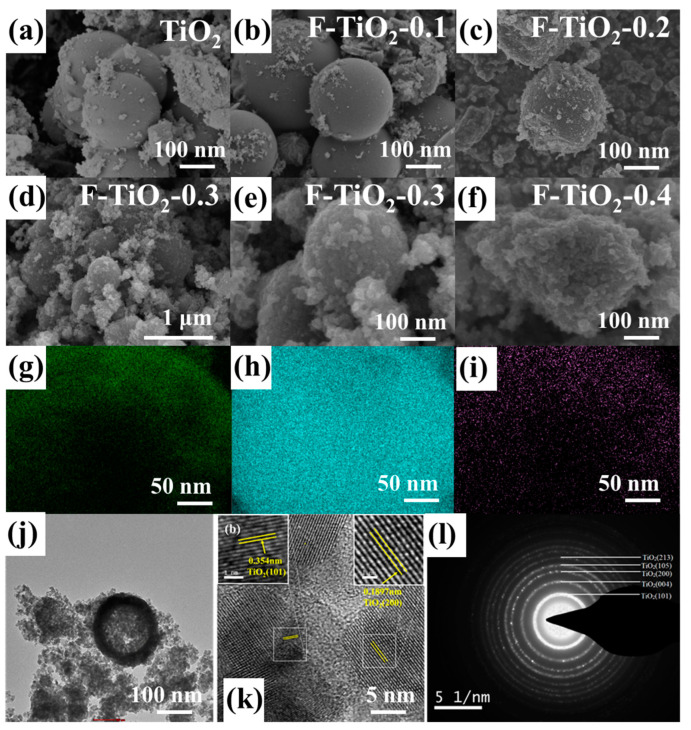
(**a**–**f**) SEM images of pure TiO_2_, F-TiO_2_-0.1, F-TiO_2_-0.1, F-TiO_2_-0.3, and F-TiO_2_-0.4 samples; (**g**–**i**) the element mapping of the O element, Ti element, and F element, respectively; (**j**,**k**) TEM image; and (**l**) SAED pattern of F-TiO_2_-0.3 hollow spheres.

Figure 3a–f shows the SEM images of pure TiO_2_ and F-TiO_2_ material prepared by hydrothermal method. The TiO_2_ sample exhibits a smooth spherical structure with a diameter of about 200–400 nm. The F-TiO_2_-0.1, F-TiO_2_-0.2, and F-TiO_2_-0.3 materials displayed in Figure 3b–f also exhibit spherical structures with diameters of about 200–400 nm. However, with the increase in hydrofluoric acid addition in the precursor solution, the surface of the F-TiO_2_ material gradually becomes rough, which favors the adsorption of gas molecules. As shown in Figure 3f, the F-TiO_2_-0.3 nanospheres with a rough surface consist of small TiO_2_ nanoparticles. When the hydrofluoric acid content in the precursor solution is further increased to the molar ratio of F to Ti elements (0.4:1), the sample of F-TiO_2_-0.4 no longer has a complete spherical structure but shows a dense packing structure, which will reduce the specific surface area of the sensing materials and is unfavorable for the adsorption of gas molecules. Furthermore, the F element contents of F-TiO_2_ samples were also investigated by the EDX spectra and are displayed in Figure S3. With the increase in hydrofluoric acid content in the precursor solution, the content of the F element in the F-TiO_2_ material also gradually increases, which also indicates that the TiO_2_ material has been successfully fluorinated. The atomic contents of Ti, O, and F elements in the F-TiO_2_-0.3 sample are about 28.18%, 68.51%, and 3.31%, respectively. In the element-mapping images (Figure 3g–i), the F element, like the O element and the Ti element, is uniformly distributed throughout the entire material. As portrayed in the TEM image in Figure 3j, the F-TiO_2_-0.3 material possesses a continuous hollow spherical morphology with approximately 54 nm-thick TiO_2_ shells. The high-resolution TEM (HRTEM) image of the F-TiO_2_-0.3 material is shown in Figure 3k. The lattice fringes with interplanar spacings of 0.354 nm and 0.1897 nm belong to the (101) and (200) lattice planes of anatase TiO_2_, respectively. Figure 3l further exhibits the selected-area electron diffraction (SAED) pattern of F-TiO_2_-0.3, where the polycrystalline diffraction rings (101), (001), (200), (105), and (213) are consistent with the anatase TiO_2_, which is in agreement with preceding XRD results.

**Figure 4:**
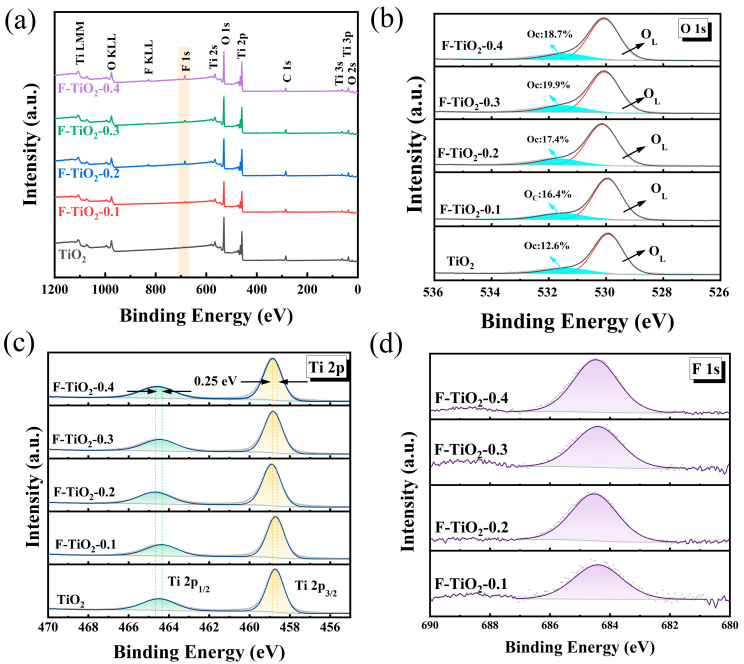
(**a**) XPS survey spectra of pure TiO_2_ and F-TiO_2_ hollow spheres; (**b**) O 1s spectra of pure TiO_2_ and F-TiO_2_ hollow spheres; (**c**) Ti 2p spectrum of F-TiO_2_ hollow spheres; and (**d**) F 1s spectra of F-TiO_2_ hollow spheres.

To further investigate the chemical composition and chemical properties of the sensing materials, the XPS spectra of pure TiO_2_ and F-TiO_2_ materials were obtained and are shown in Figure 4. As shown in the survey spectra in Figure 4a, compared with the pure TiO_2_ sample, there is an additional spectrum of the F element in the samples of F-TiO_2_, in addition to the spectra of elements Ti and O, which is due to the fluorination of the material. The O 1s spectra of pure TiO_2_ and F-TiO_2_ 2-0.3 materials are displayed in Figure 4b. The two O 1s peaks of the pure TiO_2_ material and the F-TiO_2_ materials are located at 529.97 eV and 531.67 eV, which correspond to the lattice oxygen of TiO_2_ and the adsorbed oxygen, respectively [46,47,48]. In addition, the increased oxygen content adsorbed on the surface of the F-TiO_2_ sample, compared to pure TiO_2_, is favorable for enhancing the gas-sensing properties of the material. Furthermore, it can be observed that the adsorbed content (19.9%) of the F-TiO_2_-0.3 sample is the highest. Figure 4c exhibits the characteristic peaks of Ti 2p in TiO_2_ and F-TiO_2_ samples. For the TiO_2_ sample, the two peaks corresponding to Ti-2p3/2 and Ti-2p1/2 are located at 458.17 eV and 463.92 eV, respectively. [49] The positions of Ti-2p3/2 and Ti-2p1/2 peaks in F-TiO_2_ samples shifted by 0.25 eV towards higher energy compared to pure TiO_2_, which should be related to the surface fluorination of the material. Figure 4d further exhibits F 1s peak in F-TiO_2_ samples, with a binding energy of 684.5 eV, indicating that the F element exists as surface fluoride (≡Ti-F) on the TiO_2_ material [40,50]. 

### 3.2. Electrical Measurements and Gas-Sensing Properties

**Figure 5:**
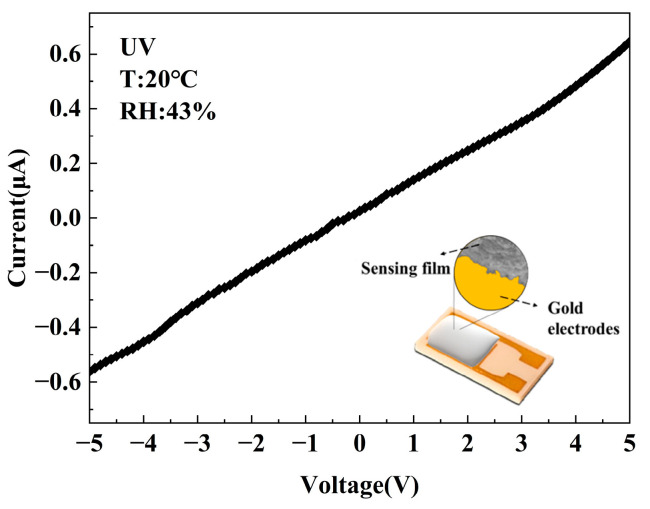
I–V characteristic curve of the F-TiO_2_-0.3 hollow sphere.

The current–voltage (I–V) polarization profile acquired from the F-TiO_2_-0.3 hollow sphere-based sensor at room temperature under UV irradiation is depicted in Figure 5, sweeping applied bias from −5 V to +5 V. It can be seen that the I–V polarization curve of the sensor shows a nearly linear trend, which means that there is an ohmic contact formed between the interdigital electrode and the fluorinated TiO_2_ materials. Therefore, the sensing signal generated by sensors based on F-TiO_2_ hollow sphere material is mainly attributed to the F-TiO_2_ sensing film.

**Figure 6:**
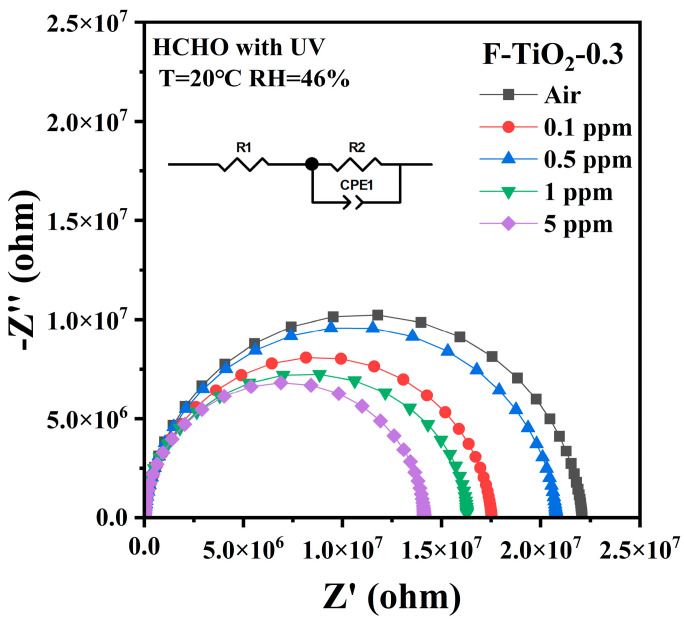
Fitting impedance spectra (1 Hz to 1 MHz) of sensors based on F-TiO_2_-0.3 hollow spheres under different environments.

To further analyze the electrical properties of the F-TiO_2_-based sensor, Figure 6 shows the fitted dielectric impedance spectra of gas sensors using F-TiO_2_-0.3 hollow spheres as sensing materials at different formaldehyde concentrations, which were tested in the frequency range of 1 Hz–1 MHz. The low-frequency intercept of the Nyquist semicircle with the real axis (Z′) constitutes the summed resistances of grain bulk and grain boundaries. Additionally, the maximum imaginary impedance (Z″) approaches half the Z′ maximum. This necessitates a constant-phase element (CPE) in the equivalent circuit whose impedance depends on CPE-T and CPE-P [51]. As P varies, the CPE can model capacitive, resistive, or inductive components [52]. The inset of Figure 6 presents the corresponding circuit, comprising bulk resistance (R_1_) and grain boundary resistance (R_2_) [53]. Upon formaldehyde exposure, the Nyquist plot radius rapidly decreases. Fitted circuit parameters at differing concentrations appear in Table 1. Both R_1_ and R_2_ decline with concentration, although R_2_ drops faster, implicating predominant grain boundary conduction [54,55]. CPE-P values around 0.97 confirm the dispersion effect on the surface, indicating excellent capacitive behavior [5,56]. CPE-T signifies capacitive changes via the depletion width modulation. Declining CPE-T indicates narrower depletion regions and an enhanced response to formaldehyde. Thus, photogenerated carrier separation in fluorinated TiO_2_ enables detection by tuning electron transport along grain boundaries.

**Figure 7:**
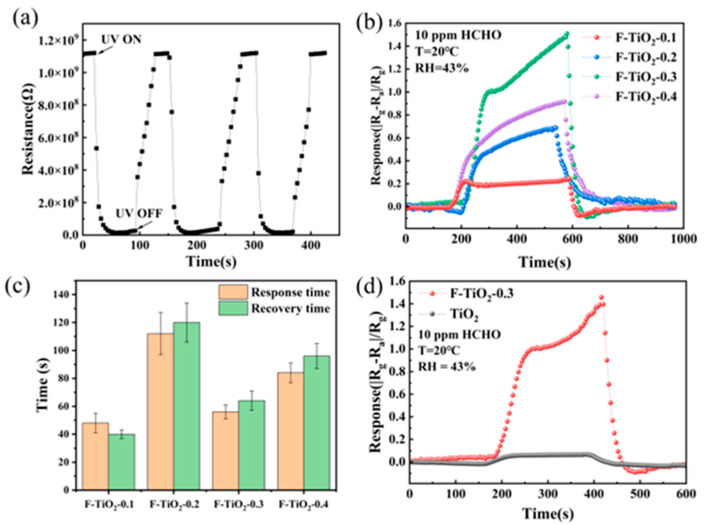
(**a**) The response curve of the F-TiO_2_-0.3 sensor to UV light; (**b**) the response curves of F-TiO_2_-0.1, F-TiO_2_-0.2, F-TiO_2_-0.3, and F-TiO_2_-0.4 sensors to 10 ppm HCHO under UV irradiation at room temperature; (**c**) response and recovery times of F-TiO_2_ sensors; (**d**) the response curves of pure TiO_2_ and F-TiO_2_-0.3 sensors to 10 ppm HCHO.

The photoresponse and recovery curves of the sensor based on F-TiO_2_-0.3 hollow spheres under UV light at room temperature were obtained and are shown in Figure 7a. Since the activation energy of UV light at a wavelength of 365 nm (used in this work) approaches the bandgap of TiO_2_ material (3.2 eV), electrons in the valence band of TiO_2_ material would be transferred to the conduction band by absorbing the energy of ultraviolet light, thus forming many photogenerated electron (e^−^) hole (h^+^) pairs in the TiO_2_ material through the following reactions [57]:

(1) $$hv\;\to h^{+}+e^{-}$$

So, as shown in Figure 7a, the F-TiO_2_-0.3 sensor had a large amplitude of resistance variation to UV light. Furthermore, the F-TiO_2_-0.3-based sensor also had a short response/recovery time (12 s/36 s) and a good repeatability to UV light. The sensor maintained a stable original resistance and a stable response resistance of 15 M Ω to UV throughout the three-cycle testing process. Figure 7b exhibits the dynamic response curves of F-TiO_2_ sensors toward 10 ppm HCHO gas under UV irradiation at room temperature. The response values of F-TiO_2_-0.1, F-TiO_2_-0.2, F-TiO_2_-0.3, and F-TiO_2_-0.4 sensors were 0.25, 0.69, 1.56, and 0.92, respectively. As the molar ratio of F to Ti content in the precursor solution increased from 0.1 to 0.3, the sensor’s response to 10 ppm formaldehyde exhibited an increasing trend. The response of the F-TiO_2_-0.3 sensor to HCHO was the highest. However, the response of the F-TiO_2_-0.4 sensor to formaldehyde gas was lower than that of the F-TiO_2_-0.3 sensor because of the destruction of the hollow spherical structure of the F-TiO_2_-0.4 sample, which is related to the decrease in the specific surface area of the F-TiO_2_-0.4 material. The F-TiO_2_-0.3 sensor also had a short response/recovery time of 56 s/64 s, as shown in Figure 7c. Furthermore, compared to the pure TiO_2_ sensor, the response to HCHO had been significantly enhanced by the formation of F-TiO_2_ hollow spheres, as shown in Figure 7d. The F-TiO_2_-0.3 sensor exhibited an approximate 18-fold enhancement in response (1.56) compared to the pure TiO_2_ sensor (0.085).

**Figure 8:**
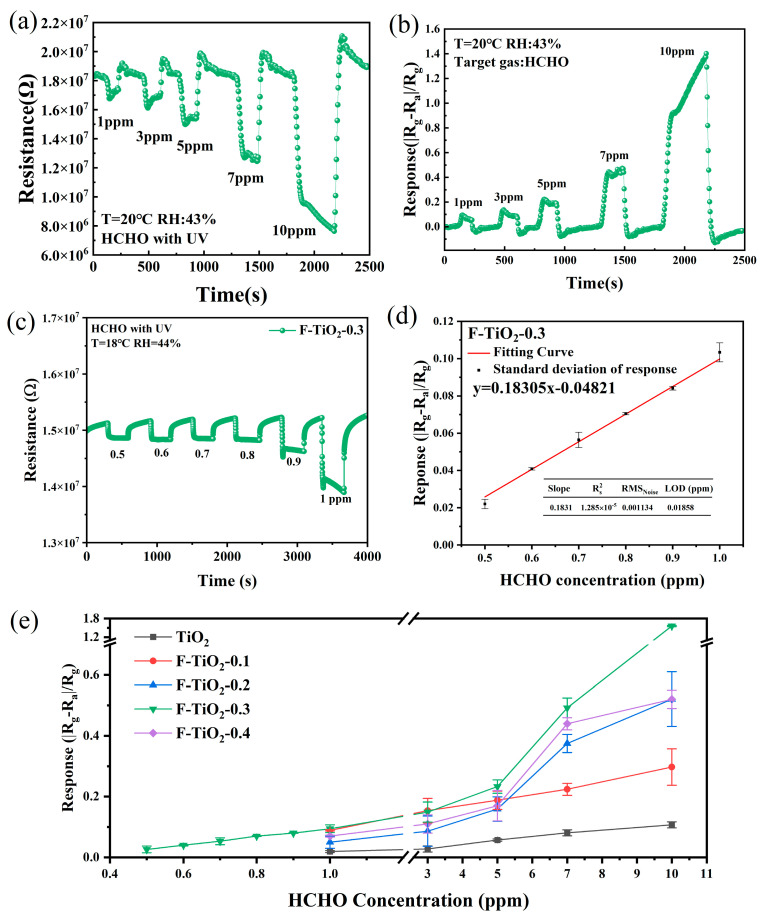
(**a**) Resistance curve of the F-TiO_2_-0.3 sensor to 1–10 ppm HCHO at room temperature under UV irradiation; (**b**) response curve of the F-TiO_2_-0.3 sensor to 1–10 ppm HCHO at room temperature under UV irradiation; (**c**) response curve of the F-TiO_2_-0.3 sensor to 0.5–1 ppm HCHO at room temperature under UV irradiation; (**d**) the fitting curve of the corresponding response values to 0.5–1 ppm HCHO gas; (**e**) the point plots of pure TiO_2_ and F-TiO_2_ sensors for 0.5–10 ppm HCHO at room temperature under UV irradiation.

Figure 8a is the dynamic resistance curve of the F-TiO_2_-0.3 sensor to 1–10 ppm HCHO gas under UV irradiation. Upon exposure to formaldehyde vapor, the resistance of the sensor decreases dramatically, indicating that the sensor has an n-type semiconductor response mechanism. With the increase in formaldehyde gas concentration, the variation range of sensor resistance increases gradually. After HCHO gas was removed, the sensor’s resistance could quickly return to its initial value. Furthermore, as displayed in Figure 8b, the F-TiO_2_-0.3 sensor’s response to HCHO gas also gradually increased as the concentration of HCHO gas increased.

The response curve of the F-TiO_2_-0.3 sensor to low concentrations of 0.5–0.9 ppm HCHO gas under UV irradiation was obtained and is shown in Figure 8c. It can be observed that the F-TiO_2_-0.3 sensor had a readily observable response to 0.5 ppm HCHO gas. The sensor could still display excellent response and recovery properties to low-concentration HCHO gas. The response curves of the F-TiO_2_-0.1, F-TiO_2_-0.2, and F-TiO_2_-0.4 sensors to 1–10 ppm HCHO under UV irradiation are displayed in Figure S4. The detection limits of these three sensors to formaldehyde gas were as low as only 1 ppm. 

Figure 8d further displays the fitted curve for the corresponding response values for 0.5–1 ppm HCHO gas, which is represented as a linear function relationship. Since the experimental conditions limit the detection limit, the theoretical limit of detection (LOD) of the F-TiO_2_-3 sensor is further analyzed based on the signal-to-noise ratio method [58]. As shown in the table inserted in Figure 8d, the resistance standard deviation (RMS_noise_) and the LOD of the F-TiO_2_-3 sensor were 0.0011 and 0.0185 ppm, respectively, which were obtained by using Formula (S2) and Formula (S3), respectively, in the Supplementary Materials. More details can be seen in Figure S5 and Table S2. The LOD of the F-TiO_2_-3 sensor was well below the minimum test concentration (0.5 ppm HCHO) in this experiment. Additionally, Figure 8e further displayed the point plots of pure TiO_2_ and F-TiO_2_ sensors for 0.5–10 ppm HCHO gas. It can be observed that among all sensors, the F-TiO_2_-3 sensor’s response was higher than that of the other sensors, especially in high formaldehyde concentrations.

**Figure 9:**
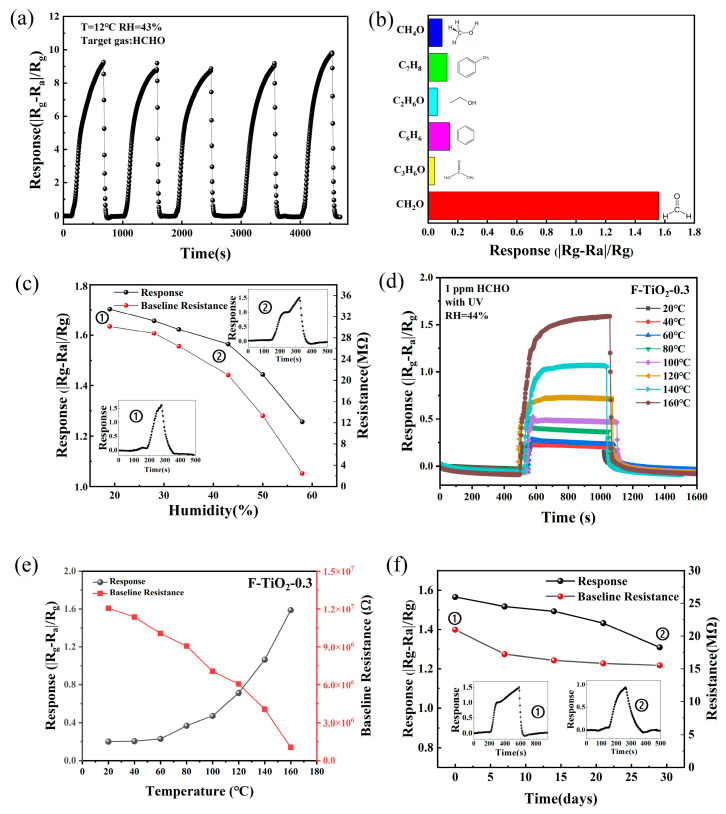
(**a**) Repeatability curve of the F-TiO_2_-0.3 sensor to 30 ppm HCHO under UV irradiation; (**b**) the response of the F-TiO_2_-0.3 sensor to formaldehyde, methanol, toluene, ethanol, benzene, and acetone at room temperature under UV light irradiation; (**c**) the response and resistance of the F-TiO_2_-0.3 sensor to 10 ppm HCHO gas under different humidity conditions; (**d**) response curves of the F-TiO_2_-0.3 sensor to 1 ppm formaldehyde at different operating temperatures under UV light activation; (**e**) response values and baseline resistances of the F-TiO_2_-0.3 sensor to 1 ppm formaldehyde at different operating temperatures under UV light activation; (**f**) the response and resistance of the F-TiO_2_-0.3 sensor to 10 ppm HCHO at room temperature under UV irradiation for 30 days.

The repeatability of the F-TiO_2_-0.3 sensor to 30 ppm HCHO gas under UV light irradiation was investigated. As shown in Figure 9a, the peak of the response values had no obvious change for five-cycle measurements. Furthermore, the response/recovery times were also analyzed and are shown in Figure S6 to be at about 100 s and 65 s, respectively. These results indicate that the F-TiO_2_-0.3 sensor had excellent signal repeatability and stability. Figure 9b shows the sensing performance of the F-TiO_2_-0.3 sensor to 30 ppm of methanol, toluene, ethanol, benzene, and acetone gas and 10 ppm of formaldehyde gas, respectively. The sensor’s response to HCHO gas was much higher than other tested analytes, indicating that the F-TiO_2_-0.3 sensor had good selectivity to HCHO. 

To probe the effect of ambient humidity on the performance of the prepared gas sensor, the F-TiO_2_-0.3 sensor was tested under different humidity conditions. As displayed in Figure 9c, the F-TiO_2_-0.3 sensor’s baseline resistance and response value for 10 ppm HCHO decreased gradually as the humidity increased from 20% to 60% because of the adsorption of H_2_O on the sensor surface, which competed with oxygen and formaldehyde molecules for active sites, thus reducing the sensing properties for formaldehyde gas [59]. Moreover, the adsorbed H_2_O molecules can transfer electrons to the sensing materials so that the F-TiO_2_-0.3 sensor displayed a decreased baseline resistance [59]. If the ambient humidity can be measured by a humidity sensor, the concentration of formaldehyde gas in the environment can be determined according to the response value of the F-TiO_2_-0.3 sensor. The influence of working temperature on the F-TiO_2_-0.3 sensor’s baseline resistance and formaldehyde-sensing performance was further investigated. As shown in Figure 9d,e, the baseline resistance and response to formaldehyde gas of the F-TiO_2_-0.3 sensor in the range of 20–160 °C were highly dependent on the working temperature. As the working temperature increases, the response value of the F-TiO_2_-0.3 sensor to 1 ppm HCHO gas gradually increases while the sensor’s baseline resistance gradually decreases, which is likely related to the fact that higher operating temperatures can increase the concentration of free electrons inside the F-TiO_2_-0.3 material and provide sufficient activation energy for the redox reactions of gas molecules. The long-term stability of the F-TiO_2_-0.3 sensor to HCHO gas was also tested and is shown in Figure 9f. The F-TiO_2_-0.3 sensor’s baseline resistance and response value for 10 ppm HCHO gas displayed a slight downward trend within 30 days, but the decrease in response value was less than 20%. After 30 days, the F-TiO_2_-0.3 sensor could also keep a high response value to HCHO gas of 1.32. Additionally, to further analyze the stability of the sensitive material in terms of chemical structure and electrical property analysis, XPS and EIS analyses of F-TiO_2_-0.3 hollow spheres after sensing tests were also obtained and are shown in Figures S7 and S8. The adsorbed oxygen content and the positions of Ti 2p, O 1s, and F 1s peaks did not change before and after the sensing tests, and the impedance spectra of the F-TiO_2_-3 sample were also almost consistent before and after the sensing tests, which further indicated that the F-TiO_2_-3 sensor has good stability. Furthermore, Table 2 shows the HCHO gas-sensing performances of the F-TiO_2_ sensor prepared in this study in comparison with the reported TiO_2_ sensors [24,60,61,62,63]. It can be observed that the F-TiO_2_-0.3 sensor has good sensing performance for HCHO gas, which performed well in terms of detection limit and response/recovery time.

### 3.3. Gas-Sensing Mechanism

**Figure 10:**
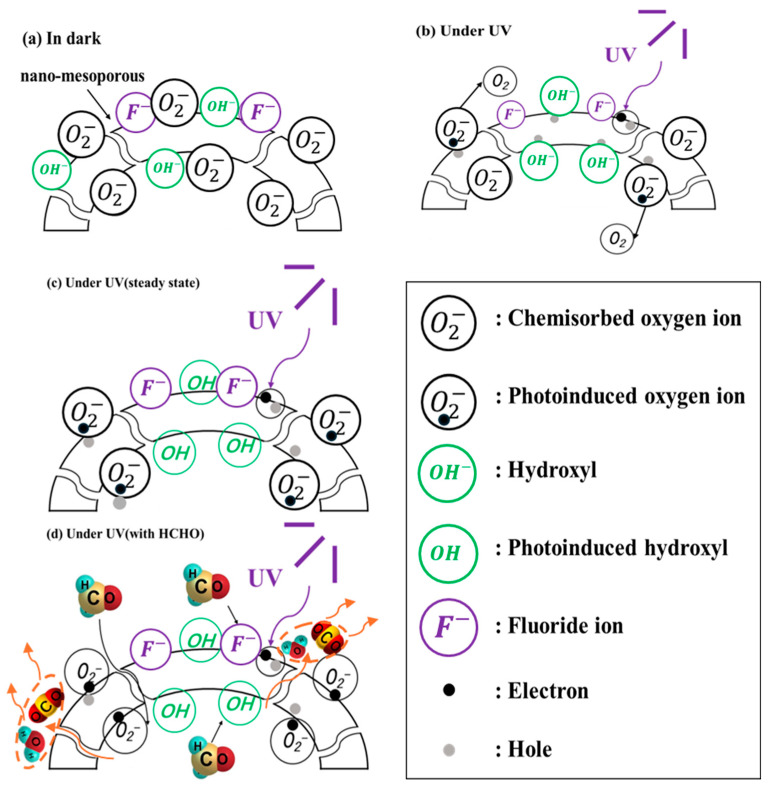
Mechanism analysis diagram of the F-TiO_2_ hollow sphere sensor in each stage.

For the F-TiO_2_ sensor, the adsorption and desorption processes of gas molecules are the key factors affecting its sensing performance. As displayed in Figure 10a, when the F-TiO_2_ sensor was exposed to air at room temperature, the physisorbed oxygen molecules ($O_{2\left(ad\right)}$) on the surface of the sensor will form chemisorbed oxygen ions ($O_{2\left(ad\right)}^{-}$) with low chemical reaction activity by trapping electrons from the conduction band of the F-TiO_2_ material, as shown in reactions (2) and (3). Furthermore, under the UV irradiation, there are many photogenerated electron (e^−^) hole (h^+^) pairs inside the F-TiO_2_ material. As shown in reactions (4) and (5), photogenerated holes will react with the chemisorbed oxygen ions, leading to oxygen ion desorption, and photogenerated electrons will also react with the physisorbed oxygen molecules, forming chemisorbed photogenerated oxygen ions $(O_{2\left(ad,hv\right)}^{-}$), which are more reactive than the chemisorbed oxygen ($O_{2}^{-}$), thus increasing the surface activity of F-TiO_2_ and enhancing the adsorption reaction of the *HOHO* gas on F-TiO_2_ [64,65]. The above adsorption and desorption processes will reach a new equilibrium state [66,67]. Furthermore, as shown in Figure 10b and reaction (6), photogenerated holes can also capture free hydroxyl groups, forming neutral *OH*∙$\left(hv\right)$ with high oxidation properties.

(2) $$O_{2\left(gas\right)}\;↔\;O_{2\left(ad\right)}$$

(3) $$e^{-}+O_{2\left(ad\right)}\;\to \;O_{2\left(ad\right)}^{-}$$

(4) $$h_{\left(hv\right)}^{+}+O_{2\left(ad\right)}^{-}\;\to \;O_{2\left(ad,hv\right)}$$

(5) $$e_{\left(hv\right)}^{-}+O_{2\left(ad\right)}\;\to \;O_{2\left(ad,hv\right)}^{-}$$

(6) $$OH^{-}+h_{\left(hv\right)}^{+}\to OH\cdot \left(hv\right)$$

As shown in Figure 10c, when *HCHO* gas is injected, *HCHO* molecules react with the $O_{2\left(ad,hv\right)}^{-}$ and *OH*∙$\left(hv\right)$, which have high oxidation properties (reaction (7)), forming *CO*_2_ and *H*_2_*O* and releasing the electrons trapped by $O_{2\left(ad,hv\right)}^{-}$ to the sensing materials, thus leading to a decrease in sensor resistance [3].

(7) $$4HCHO+4OH\cdot \left(hv\right)+3O_{2}^{-}\left(hv\right)\to \;4CO_{2}+6H_{2}O+3e^{-}\;$$

Moreover, the F-TiO_2_ sensor’s exceptional sensing properties are also related to the following factors. Firstly, the surface fluoride (≡Ti−F) with strong polarity modified on the surface TiO_2_ hollow spheres has a strong adsorption ability for electrons and can impede the recombination of the photogenerated electron hole pairs [28]. The electrons trapped by the ≡Ti−F are then further transferred to the adsorbed oxygen on the material surface [29]. Furthermore, because the *OH*∙$\left(hv\right)$ free radicals generated on the F-TiO_2_ material surface have greater mobility and stronger oxidizing ability than those generated on the surface of pristine TiO_2_, the oxidation reaction of formaldehyde molecules exhibited in reaction (7) was promoted, thus enhancing the sensing response to *HCHO* gas [3,39,68]. Secondly, the hollow sphere structure of the F-TiO_2_ material endows it with an elevated specific surface area, furnishing abundant active sites for the adsorption of formaldehyde molecules and $O_{2}^{-}\left(hv\right)$ so that more formaldehyde molecules can participate in the sensing reactions.

## 4. Conclusions

In summary, the F-TiO_2_ hollow spheres with varying ratios of fluorine to titanium were synthesized through a simple hydrothermal process by controlling hydrofluoric acid content in the hydrothermal reaction reagent. The morphology, structure, and chemical properties of the F-TiO_2_ materials had been verified by use of SEM, TEM, XRD, and XPS. The F-TiO_2_ hollow spheres sensor displayed commendable sensing properties to formaldehyde gas at room temperature under UV irradiation. The F-TiO_2_-0.3 sensor demonstrated an 18-fold higher response value (1.56) than that of the pristine TiO_2_ sensor for 10 ppm HCHO gas. The response/recovery time of the F-TiO_2_-0.3 sensor for 10 ppm HCHO is about 56 s/64 s. Furthermore, at room temperature, the F-TiO_2_-0.3 sensor also presented a low detection limit, good stability, and high selectivity to HCHO gas, indicating a good application prospect in HCHO of 0.5–10 ppm monitoring. To meet the cross-sensitivity requirements of the World Health Organization for formaldehyde sensors, in future research, precious metals (Au, Pt, Pd, etc.) or other metal oxides will be introduced to construct F-TiO_2_-based heterojunction-sensing materials to further promote the sensing properties of F-TiO_2_ sensors for low-concentration formaldehyde gas. Additionally, the negative impact of high-humidity conditions on formaldehyde-sensing properties cannot be ignored. So, in future research, materials with high moisture resistance could be introduced, such as SnTe material developed by our team recently [14] to improve the moisture resistance of F-TiO_2_-based sensors.

## Figures and Tables

**Table 1:** Equivalent fitting circuit parameter table of F-TiO_2_-0.3 hollow sphere-based gas sensor with respect to various formaldehyde concentrations.

HCHO Conc.	R_1_	R_2_	CPE1-T	CPE1-P
Air	5235	2.21 × 10^7^	2.46 × 10^−11^	0.977
0.5 ppm	5187	2.08 × 10^7^	2.27 × 10^−11^	0.961
1 ppm	5123	1.73 × 10^7^	1.82 × 10^−11^	0.960
5 ppm	5098	1.60 × 10^7^	1.47 × 10^−11^	0.973
10 ppm	4892	1.48 × 10^7^	9.35 × 10^−12^	0.959

**Table 2:** The HCHO gas-sensing performances of the F-TiO_2_ sensor prepared in this study in comparison with the reported TiO_2_ sensors.

Sensing Material	Temp. (°C)	Res.	Conc. (ppm)	Detection Limit (ppm)	Res./Recov. Time (s)	Ref.
TiO_2_ nanofibers	RT	1.41	10	10	135/85	60
CQD/TiO_2_ nanofibers	RT	1.62	0.5	0.5	45/60	60
MIP/TiO_2_ NTA	RT	13%	1	1	300/300	61
TiO_2_ (nanotube)	RT	2.3	50	10–50	180/120	62
Au/TiO_2_	RT	8.5	5	0.1	36/110	24
SnO2@TiO2	240	18.3	100	5	13/9	63
F-TiO_2_-0.3	RT	1.56	10	0.5 (LOD: 0.0185 ppm)	56/64	This work

## Data Availability

Data are contained within the article and Supplementary Materials.

## Supplementary Materials

The following supporting information can be downloaded at: https://www.mdpi.com/article/10.3390/ma17040904/s1, Figure S1: The static testing system used for testing the gas sensing performance of the sensors; Figure S2: The response of the F-TiO_2_-0.3 based sensor to different concentrations of water molecules; Figure S3: The EDX spectra and atomic content of each element of pure TiO_2_, F-TiO_2_-0.1, F-TiO_2_-0.1, F-TiO_2_-0.3, and F-TiO_2_-0.4 samples; Figure S4: Resistance curves of (a) F-TiO_2_-0.1 sensor, (b) F-TiO_2_-0.2 sensor, and (c) F-TiO_2_-0.4 sensor to 1-10 ppm HCHO at room temperature under UV irradiation; Figure S5: 5th order polynomial fit of the F-TiO_2_-0.3 sensor at the baseline before HCHO exposure; Figure S6: Response and recovery times of F-TiO_2_-0.3 sensor to 30 ppm HCHO under five repeatability tests; Figure S7: (a) O 1s spectra, (b) Ti 2p spectra, and (c) F 1s spectra of F-TiO_2_-0.3 hollow sphere before and after the sensing tests; Figure S8: Fitting impedance spectra of F-TiO_2_-0.3 hollow sphere sensor before and after the sensing tests; Table S1: Composition of the aqueous solutions used for preparation of the formaldehyde vapors; Table S2: 5th order polynomial fitting data of the F-TiO_2_-0.3 based sensor.
