# Supplementary material for: Fluorinated TiO2 Hollow Spheres for Detecting Formaldehyde under UV Irradiation
Source: Materials (Basel). 2024 Feb 15;17(4):904. doi: 10.3390/ma17040904 (PMC10889930; doi:10.3390/ma17040904)
Supplement: Supplementary file 1 [file materials-17-00904-s001.zip › materials-2770621-supplementary.pdf]

materials

MDPI

Supporting Information

Fluorinated TiO_2 Hollow Spheres for Detecting Formaldehyde under UV Irradiation

Jianwei Zhang [1], Baoyu Huang [2], Xinlei Li [2], Chao Yang [3], Wenzhuo Zhao [2], Xiuhua Xie [4], Nan Wang [2] and Xiaogan Li [2,*]

1 School of Artificial Intelligence, Dalian University of Technology, Dalian 116024, China; jwzhang@dlut.edu.cn (J.Z.)
2 School of Integrated Circuits, Dalian University of Technology, Dalian 116024, China; huangby@dlut.edu.cn (B.H.); lixinlei96@163.com (X.L.); 13193718736@163.com (W.Z.); wang_nan@dlut.edu.cn (N.W.)
3 Beijing Research Institute of Telemetry, Beijing 100076, China; ycbj220@126.com
4 State Key Laboratory of Luminescence and Applications, Changchun Institute of Optics, Fine Mechanics and Physics, Chinese Academy of Sciences, No. 3888 Dongnanhu Road, Changchun 130033, China; xiexh@ciomp.ac.cn
* Correspondence: lixg@dlut.edu.cn

Citation: Zhang, J.; Huang, B.; Li, X.; Yang, C.; Zhao, W.; Xie, X.; Wang, N.; Li, X. Fluorinated TiO_2 Hollow Spheres for Detecting Formaldehyde under UV Irradiation. *Materials* **2024**, *17*, 904. https://doi.org/10.3390/ma17040904

Academic Editors: Daniela Iannazzo and Albena Paskaleva

Received: 28 November 2023
Revised: 5 February 2024
Accepted: 9 February 2024
Published: 15 February 2024

Copyright: © 2024 by the authors. Submitted for possible open access publication under the terms and conditions of the Creative Commons Attribution (CC BY) license (https://creativecommons.org/licenses/by/4.0/).

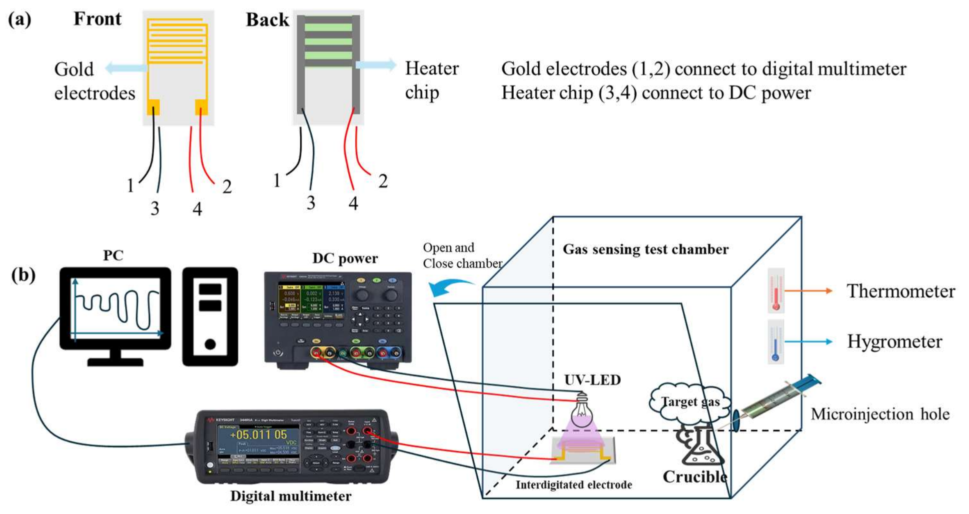

Figure S1. The static testing system used for testing the gas-sensing performance of the sensors.

The interdigital electrode used in this work to make the sensor is shown in the top part of Figure S1. The gold electrode was printed on the Al_2O_3 substrate by screen printing. The interdigital electrode is connected to the multimeter through wires 1 and 2 to measure the sensor resistance. The heater chip on the back of the Al_2O_3 substrate is connected to DC power through wires 3 and 4 to heat the electrode.

The static testing system used for testing the gas-sensing performance of the sensors in this work is shown in the bottom part of **Figure S1**. During the test process, the liquid reagent was injected into the evaporator in the test chamber by the microinjector through the sealed inlet ports to obtain the corresponding target gas. The solution volume corresponding to the concentration of the target gas is calculated by the following formula:

$$c=\frac{n*22.4}{50}=\frac{\frac{m}{M}*22.4}{50}=\frac{\frac{\rho*v*w\%}{M}}{50}*22.4 \quad \text{(S1)}$$

where *C* (ppm) represents the concentration of the target gas, ϱ is the density of the corresponding solvent, *v* (μL) is the volume of the corresponding solvent, w% is the mass fraction of solute in solvent, *n* represents the amount of substance, and *M* stands for molar mass.

After the test is completed, open the test chamber to allow the test gas inside to be discharged. The thermometer and hygrometer inside the testing chamber are used to monitor the ambient temperature and humidity during the testing process. In the experiment, the ambient humidity was maintained at about 43±2% and the temperature was maintained at about 20±2 °C.

For pure solute solutions including ethanol, methanol, acetone, benzene, and toluene (99.9% wt.%), only high-purity target gases can be obtained by evaporating the corresponding liquid reagent. However, for non-pure solute solutions including formaldehyde (40 wt.%), evaporation of the solution not only obtains the target gas but also produces water molecules. As shown in **Table S1**, injecting 10 ppm formaldehyde into the test chamber will introduce 24.7 ppm water molecules; the response properties of the F-TiO_2-0.3-based sensor to different concentrations of water molecules have been studied (**Figure S2**). It can be seen that no obvious response signal was observed. Compared with formaldehyde gas, the water produced by solution evaporation will not interfere with the performance of the sensor.

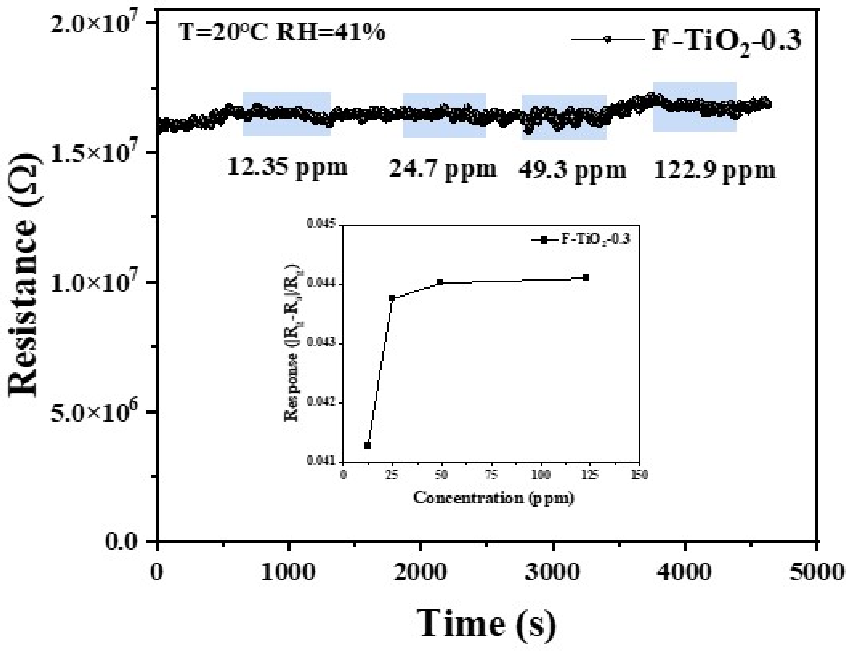

Figure S2. The response of the F-TiO_2-0.3-based sensor to different concentrations of water molecules.

Table S1. Composition of the aqueous solutions used for preparation of the formaldehyde vapors.

**Targeted HCHO (ppm)**	**5**	**10**	**20**	**50**
Total volume of HCHO solution (μL)	0.82	1.65	3.3	8.2
The volume of the corresponding H_2O (μL)	0. 5	0.99	1.98	4.94
Concentrations of the corresponding H_2O (ppm)	12.35	24.7	49.3	122.9

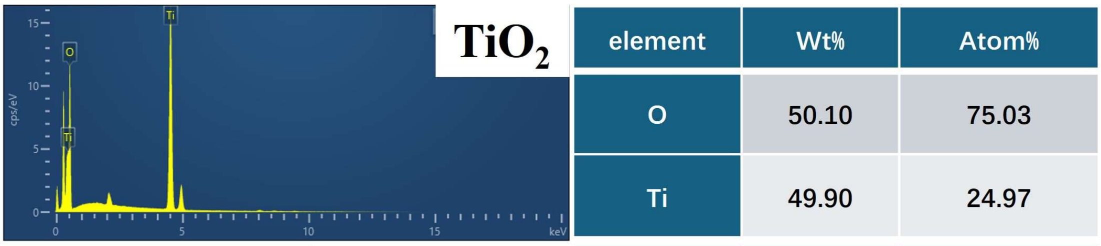

element	Wt%	Atom%
O	50.10	75.03
Ti	49.90	24.97

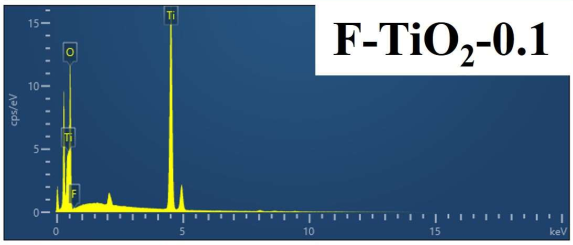

element	Wt%	Atom%
O	46.79	71.79
Ti	52.00	26.65
F	1.21	1.56

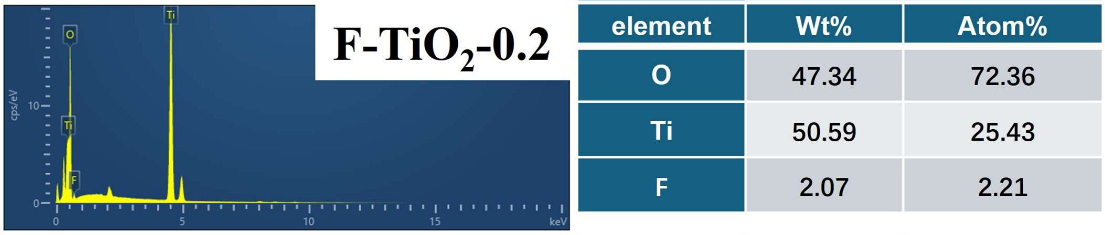

element	Wt%	Atom%
O	47.34	72.36
Ti	50.59	25.43
F	2.07	2.21

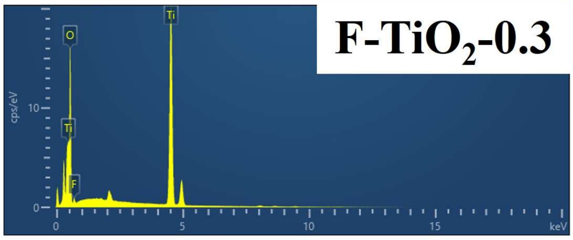

element	Wt%	Atom%
O	43.70	68.51
Ti	53.80	28.18
F	2.50	3.31

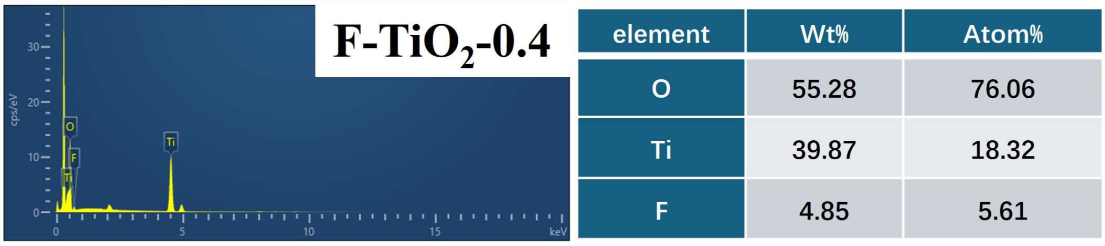

element	Wt%	Atom%
O	55.28	76.06
Ti	39.87	18.32
F	4.85	5.61

Figure S3. The EDX spectra and atomic content of each element of pure TiO$_2$, F-TiO$_2$-0.1, F-TiO$_2$-0.1, F-TiO$_2$-0.3, and F-TiO$_2$-0.4 samples.

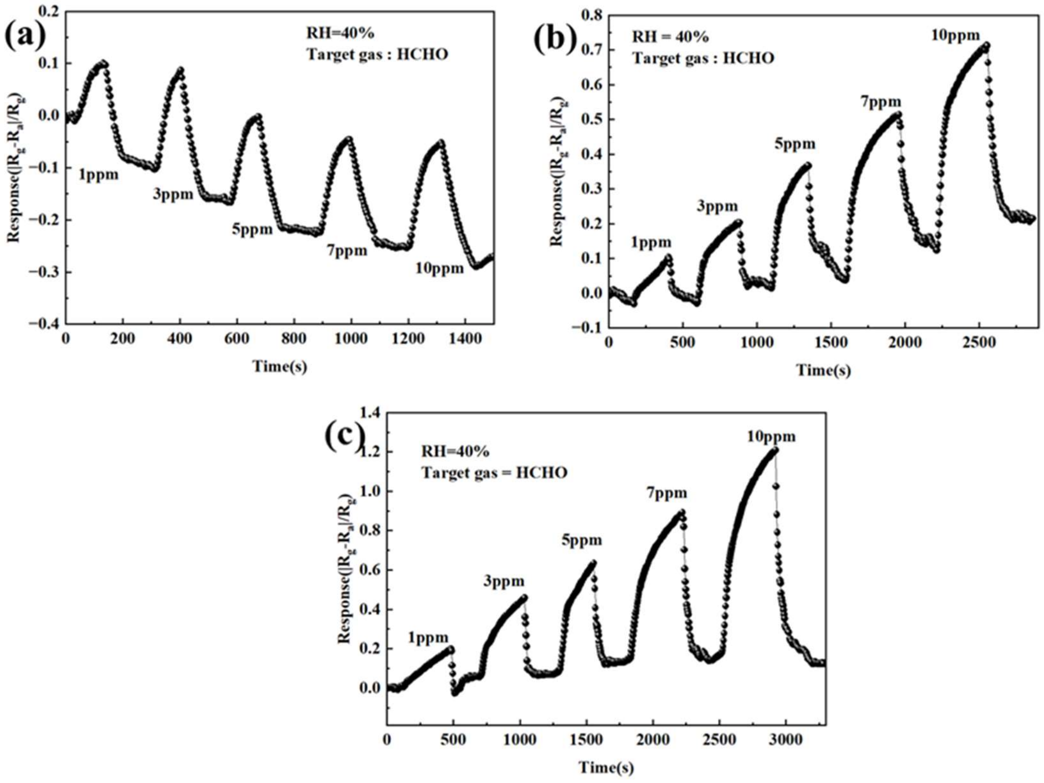

Figure S4. Resistance curves of (a) the F-TiO_2-0.1 sensor, (b) the F-TiO_2-0.2 sensor, and (c) the F-TiO_2-0.4 sensor to 1–10 ppm HCHO at room temperature under UV irradiation.

The LOD can be estimated by following equations (S2) and (S3) according to the signal-to-noise approach (Li et al., *Nano Letters 2003*, 3, **7**, 929–933):

$$\mathrm{LOD} = 3 \times \frac{RMS_{noise}}{b} \tag{S2}$$

$$RMS_{noise} = \sqrt{\frac{\sum_{i=1}^{n}\left(R_i - \overline{R}\right)^2}{n}} \tag{S3}$$

where RMS_{noise} (root mean square noise) is the standard deviation of resistance (R), R_i is the measured resistance, $\overline{R}$ is the average of R_i, and b is the slope in the calibration curve between the sensor response and gas concentrations. In this work, the RMS_{noise} of the F-TiO_2-0.3 sensor is calculated to be 0.0011 and the slope (b) of the sensor is 0.0482, as shown in Fig. 8(d). So, the LOD of the F-TiO_2-0.3 sensor is estimated to be 0.0185 ppm.

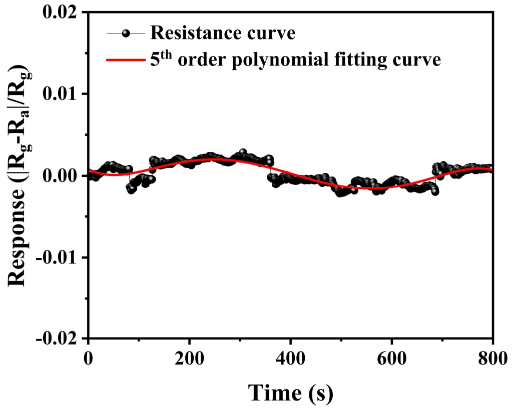

Figure S5. Fifth-order polynomial fit of the F-TiO_2-0.3 sensor at the baseline before HCHO exposure.

Table S2. Fifth-order polynomial fitting data of the F-TiO_2-0.3-based sensor.

Time	R_i-$\bar{R}$	$(R_i$-$\bar{R})^2$
0	5.21E-04	2.71321E-07
80	-3.99E-05	1.5924E-09
160	1.04E-03	1.09122E-06
240	1.70E-03	2.90571E-06
320	1.25E-03	1.57406E-06
400	-1.64E-05	2.69449E-10
480	-1.34E-03	1.78326E-06
560	-1.88E-03	3.51707E-06
640	-1.23E-03	1.51725E-06
720	1.25E-04	1.56115E-08
800	4.18E-04	1.7451E-07

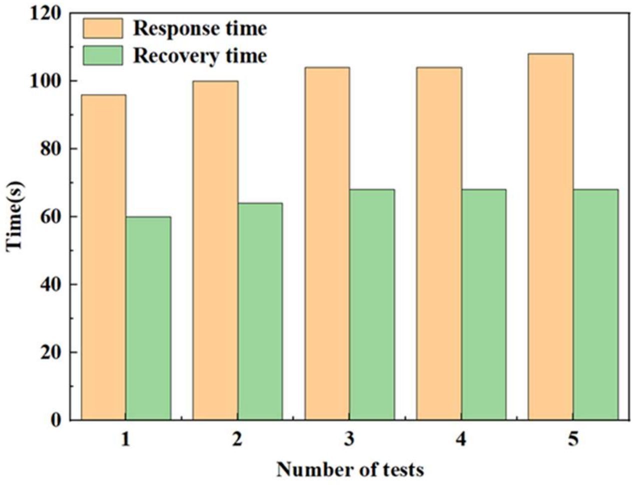

Figure S6. Response and recovery times of the F-TiO_2-0.3 sensor to 30 ppm HCHO under five repeatability tests.

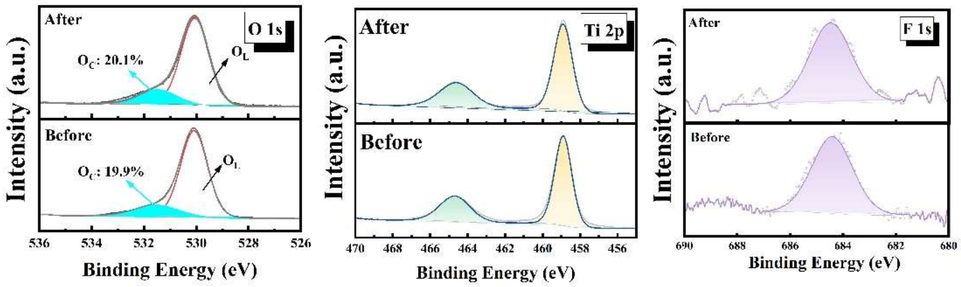

Figure S7. (a) O 1s spectra, (b) Ti 2p spectra, and (c) F 1s spectra of F-TiO_2-0.3 hollow spheres before and after the sensing tests.

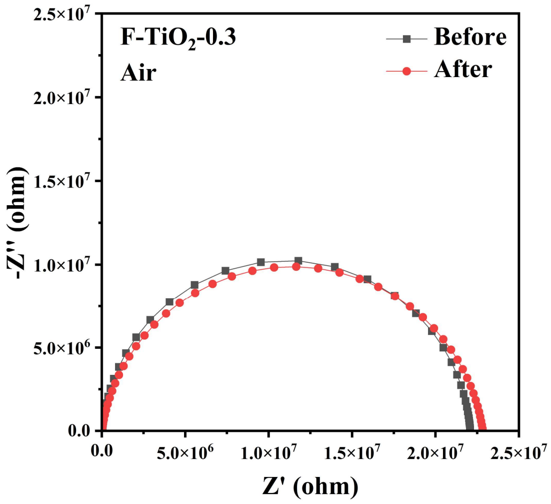

Figure S8. Fitting impedance spectra of the F-TiO_2-0.3 hollow sphere sensor before and after the sensing tests.
